# Graphene Oxide: From Tunable Structures to Diverse Luminescence Behaviors

**DOI:** 10.1002/advs.201900855

**Published:** 2019-06-14

**Authors:** Qingsong Mei, Bianhua Liu, Guangmei Han, Renyong Liu, Ming‐Yong Han, Zhongping Zhang

**Affiliations:** ^1^ School of Food and Biological Engineering Hefei University of Technology Hefei Anhui 230009 China; ^2^ CAS Center for Excellence in Nanoscience Institute of Intelligent Machines Hefei Anhui 230031 China; ^3^ School of Chemistry and Chemical Engineering Anhui University Hefei Anhui 230601 China

**Keywords:** chemiluminescence, electroluminescence, fluorescence, graphene oxide, tunable structures

## Abstract

Since the first discovery of luminescent graphene oxide (GO), exponentially increasing investigations on the tunable structures and surfaces for modulating its optical properties have struggled to expand applications in imaging, sensing, biomedical diagnostics, and so on. Here, the latest works on reconstructing or modifying the structures and surfaces of GO to achieve diverse luminescence are systematically reviewed, including fluorescence, electroluminescence, and chemiluminescence. Moreover, the fundamental difficulties of the investigations and applications of luminescent GO nanomaterials are clarified to inspire more constructive thoughts for expanding their application boundaries.

## 1 Introduction

In 2004, the appearance of atomically thin graphene nanosheets through peeling off highly oriented pyrolytic graphite completely reversed scientists' beliefs that single‐layer carbon atom structure was thermodynamically unstable because it was prone to form curved structures such as fullerenes and nanotubes under ambient conditions.^1^ Due to its extraordinary physical properties, this revolutionary 2D material was then awarded the 2010 Nobel Prize in physics, and ignited tremendous and increasing research interests in the fields of physics and material science over the last decade.^2^ Moreover, graphene also provides chemists with a huge imaginary space to create novel carbon atoms–packed materials, 2D graphene derivatives, such as nitrogen‐ or boron‐doped graphene, graphdiyne, graphane, and so on.^3^ The fascinating chemical/physical properties of these materials reinspire researchers with intense interests in graphene oxide (GO), a typical graphene derivative that actually has been discovered by Brodie through oxidizing flake graphite in nitric acid and potassium chlorate as early as 1859.^4^ Like graphene, GO possesses a large rigid π‐conjugated planar structure, coupling with carboxyl groups on the edge and hydroxyl/epoxy groups on basal plane,^5^ which provides various possibilities to reconstruct the structure and surface of GO for the creation of new physicochemical properties toward the potential applications in biological imaging or sensing, drug delivery, composite materials, and luminescent devices.

Introducing oxygen‐containing groups into graphene lattice makes GO nanosheets exhibit many amazing properties, such as excellent water dispersibility, surface modifiability, biological compatibility, and so on.^6^ The most notable is that the infinite large sp^2^ domain is disrupted after oxidizing, and the bandgap can be opened to transform the nonluminescent graphene into luminescent GO. However, the epoxy and carboxyl groups on GO usually induce nonradiative recombination of localized electron–hole (e–h) pairs. Thus, the traditional GO remains to keep its nonemissive property.^7^ Moreover, Lu et al. first reported the highly efficient fluorescence‐quenching ability of GO to a variety of fluorophores by the energy transfer mechanism,^8^, ^9^ which has been widely used to design fluorescent sensors for the detection and imaging of analytes.^10^, ^11^, ^12^, ^13^, ^14^, ^15^, ^16^, ^17^, ^18^ This universal sensing platform should be attributed to its rigid π‐conjugated planar structures for easily anchoring fluorescence donors and detection targets, and its widespread absorbance spanning from visible to near‐infrared (NIR) region.^19^, ^20^

With increasing understanding on GO, various methods have been developed to appropriately modulate its structures, and meanwhile, the diverse luminescence behaviors are also observed by photon or electron excitation. Typically, chemical modification may break its large π‐conjugated network to form numerous isolated sp^2^ domains on the rigid plane, and facilitate the radiative recombination of localized e–h pairs, thus giving out various luminescence. On the other hand, oxidative or reductive cutting of GO nanosheets into 0D quantum dots (QDs) can further create discrete energy levels. In the past decade, our group has focused on the investigations of structural tunability and luminescent properties of GO. For example, we have proposed an alkylamine modification method to greatly improve GO luminescent quantum yield for the first time,^7^ developed a novel peroxidation strategy to enrich GO luminescent colors,^21^ and recently found that ultrastrong chemiluminescence (CL) could be initiated by GO.^22^ Additionally, a series of progresses on luminescent GO nanomaterials and their sensing applications have been made during these years.^23^

In early stage of luminescent GO investigations, Loh et al. published the first review that has highlighted the chemical platform of GO for optical applications.^24^ Later, Cao et al. have summarized the fluorescent properties of graphene versus other carbon materials.^25^ Additionally, the biomedical applications of graphene and GO, such as fluorescence bioimaging and cancer therapy, have been reviewed.^26^ However, the recent advances on GO urgently need a systematical summary to account for the relationships between tunable structures and diverse luminescence behaviors, and prospect the future breakthrough in fundamental and applicable explorations. In this review, we first introduce several classical structural models of GO and the modulation methods for its morphology sizes, surface functionalities, as well as its bandgap energies, then summarize the latest progresses on the diverse luminescent behaviors from GO and its derivatives, including photoluminescence (PL), electroluminescence (EL), and CL, and finally give future perspectives, hoping to offer a full‐scale insight to help the further exploration of exceptional emissive GO nanomaterials, and expand their application boundaries.

## 2 Structural Diversities of GO

To preferably modify GO nanostructures, it is of great importance to give a comprehensive insight into its chemical structure. However, the structure of GO varies in size, morphology, and surface/lateral groups, which depends on its preparation protocols. In general, preparation of GO from graphite mainly uses three typical protocols employing flake graphite as initial material.^27^, ^28^ It can be traced back to the year of 1859 that Brodie discovered the formation of yellow graphitic acid after oxidizing flake graphite in nitric acid and potassium chlorate, and found that the obtained material could be dispersed in pure or basic water.^4^, ^29^ After that, Staudenmaier optimized Brodie's preparation procedure by use of concentrated H_2_SO_4_ and HNO_3_, minimizing explosion risks caused by the accumulation of ClO_2_.^30^ Hummers and Offeman developed an alternative oxidation method by reacting graphite with a mixture of potassium permanganate and concentrated H_2_SO_4_.^31^ This procedure can be applied on a multigram scale and becomes the most frequently used method to prepare GO. Though others have developed slightly modified preparation protocols, these three methods mentioned above comprise the primary routes for synthesis of GO.

### 2.1 Chemical Structures of GO

Since the first preparation by oxidizing graphite flakes, studies on GO structural models have never been terminated.^32^ Up to date, various models have been proposed based on the molecular dynamics simulations, first‐principle calculations, and experimental characterizations.^33^ However, because GO is not stoichiometric and nearly amorphous, its precise chemical structure is still uncertain and may vary with the synthesis methods and environment factors. In this review, we briefly summarize the proposed models for clearly understanding the chemical treatment protocols of luminescent GO which is introduced hereinafter.

Originally, Hofmann and Holst proposed that oxygen atoms were bound to carbon atoms of the hexagon layer planes by epoxy linkages with an ideal formula of ether bonds.^34^ However, this model does not contain any hydrogen atoms that are indeed existed in GO nanosheets. Ruess then assumed that a wrinkled carbon network is composed of *trans*‐linked cyclohexane chairs, and tetrahedrally coordinated carbon atoms were bound to axial hydroxyl groups.^35^ This is the first model to account for the hydrogen content of GO nanosheets, while does not contain C=O bonds. Clauss and co‐workers then modified this model with carbon double bonds, ketone, and enolic groups, and explained that the observed acidity of GO should be ascribed to enolic hydroxyl species and carboxylic groups on the GO nanosheets.^36^ After stereochemical reconsiderations, this model was revised by Scholz and Boehm.^37^ They assumed a keto/enol tautomerism in GO, and the enol form endowed the acidic sites. Szabo et al. revised the Scholz model, and proposed a corrugated carbon skeleton consisting of two kinds of regions, *trans*‐linked cyclohexane chairs, and ribbons of flat hexagons with carbon double bonds and functional groups such as tertiary hydroxyl, 1.3‐ether, ketone, quinone, and phenol.^38^

**Figure 1:**
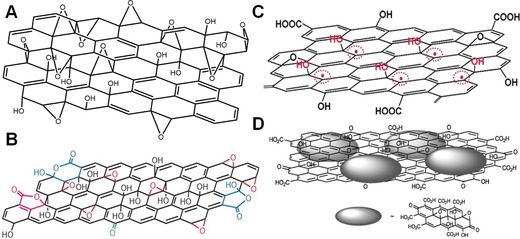
A) The Lerf–Klinowski structural model of GO nanosheet. Reproduced with permission.^38^ Copyright 2006, American Chemical Society. B) An updated model proposed by Gao et al. Reproduced with permission.^41^ Copyright 2009, Springer Nature. C) π‐conjugated carbon radical model of GO nanosheet. Reproduced with permission.^22^ Copyright 2014, Wiley‐VCH. D) GO nanosheets structural model with surface‐bound oxidative debris. Reproduced with permission.^44^ Copyright 2011, Wiley‐VCH.

Based on nuclear magnetic resonance spectroscopy (NMR) studies, Lerf and co‐workers depicted a GO layer as a random distribution of flat aromatic regions with unoxidized benzene rings and wrinkled regions of alicyclic six‐membered rings bearing carbon double bonds, hydroxyl and ether groups, and the sheets of GO terminated with hydroxyl and carboxylic groups.^39^ This model is named Lerf–Klinowski model (**Figure** **1**A), and is widely adopted in literatures. Cai et al. also found that hydroxyl groups bonded to carbon atom accompanying with epoxy groups bonded to neighboring carbon atoms through NMR studies.^40^ Ajayan and co‐workers considered the Lerf–Klinowski model as base structure, and further offered experimental support for a peripheral structure containing five‐ and six‐membered ring lactols and possibly an occasional 2‐hydroxynaphthalic anhydride or 1,3‐dihydroxyxanthone (Figure 1B).^41^ They believed that the presence of numerous tertiary alcohols in GO allowed for the possibility of reacting with nearby carboxylic acids either on the same GO sheet or an adjacent sheet to generate an ester. By use of ultrahigh‐resolution transmission electron microscopy images, Zettl and co‐workers found that GO nanosheets were highly inhomogeneous. They believed that hydroxyls and epoxies were the dominant functionalities because only these oxidative functionalities would result in fully restoring graphitic character once reduced, as other high energy peroxide functionalities would induce C—C bond breakage.^42^ Additionally, based on first‐principle calculations, Li et al. proposed an unzipping mechanism, where the epoxy groups were suggested to have a preference for aligning in a line. The aligned epoxy groups then induced a rupture of the underlying C—C bonds.^43^

The research about chemical structures of GO does not end there due to many arising phenomena which existing structure models cannot explain. Recently, our group provided a novel understanding of the reaction mechanism for synthesis of GO and new insight into its chemical structure.^22^ We believed that a large number of π‐conjugated carbon radicals were formed by the addition of hydroxyl radicals to the carbon double bonds on the disrupted π‐network plane of GO (Figure 1C). In addition, another recent model believed that GO consisted of two different components: one was graphene with a low degree of oxidation, while the other was oxidative debris (OD) of graphite which was a polyaromatic oxide material and was produced during the oxidative transformation of graphite into GO (Figure 1D).^44^ After the purification process, OD was claimed to be only partially removed with about 30% of the GO weight, and the residual OD was randomly adhered to the surface of GO nanosheets.

### 2.2 Morphological Diversity and Functionalization of GO

The synthesis strategies not only affect functional groups of GO nanosheets, but also change their morphologies. Dependent on the reacting conditions, such as starting graphite materials, types of oxidants, and sonication times, the obtained GO could exhibit as large and flat nanosheets with a lateral size of hundreds of nanometers, or 0D QDs with diameters of several nanometers. After transformation of carbon double bonds in graphite layers to various oxygenated groups, three major structural features might be presented in the nanosheets: holes within the basal plane caused by the evolution of carbon atoms into gaseous CO or CO_2_, graphitic sp^2^ regions, and sp^2^/sp^3^ mixed regions.^45^ Under more harsh synthesis conditions, for example, utilization of ultrastrong oxidants, sonicating for long times, and so on, the sp^2^‐hybridized carbon networks will break down into small fragments.

Furthermore, the strong π‐conjugated network and abundant oxygen‐containing species on GO nanosheets facilitate the modifications with various targets. DNA, peptides, conjugated polymer chains can be adsorbed onto GO surface through noncovalent π–π interactions or electrostatic interactions. Many organic molecules are able to covalently conjugate with GO. For example, carboxyl groups at the edge of GO nanosheets can react with amino groups or hydroxyl groups through acyl reactions, epoxy groups in GO planes also provide active sites for ring‐opening reactions with amino groups, adjacent dihydroxy groups react with borate compounds.^5^ These structural variations, including morphological sizes and covalent functionalization of GO nanomaterials, greatly modulate their properties, such as surface hydrophilicity, organelle targetable ability, and especially tune the bandgap energies through controlling the size of sp^2^ domains on GO nanosheets.

### 2.3 Bandgap Modulation of GO

**Figure 2:**
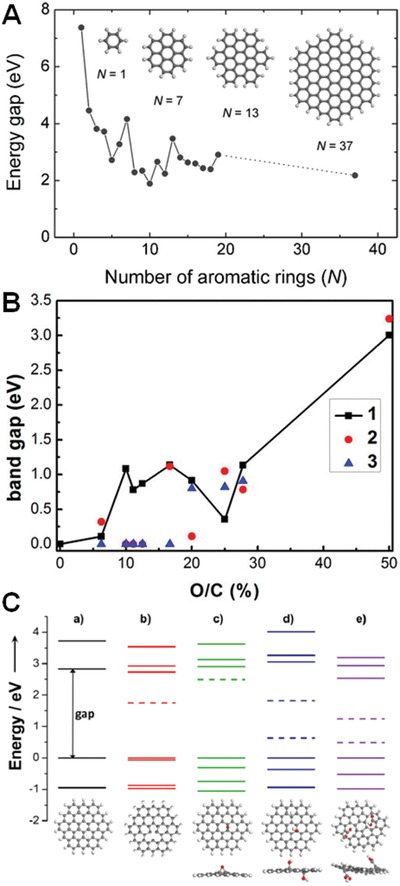
A) Energy gap of π–π* transitions as a function of the fused aromatic ring numbers. Reproduced with permission.^46^ Copyright 2010, Wiley‐VCH. B) The bandgap of reduced GO versus the O/C ratios. The squares, circles, and triangles corresponded to the most stable, second most stable, and the third most stable configurations of each O/C ratio, respectively. Reproduced with permission.^48^ Copyright 2012, American Institute of Physics. C) The energy levels of ideal graphene nanosheets and decorated with various functional groups such as an atomic vacancy, hydroxyl, epoxy, and carboxyl groups. Reproduced with permission.^49^ Copyright 2012, Wiley‐VCH.

Although the exact structure of GO remains uncertain, it is well accepted that the bandgap is highly dependent on their morphology sizes, oxygen coverage densities of O/C ratio and surface functionalities, and so on. First, the numbers of conjugated aromatic rings increase with the size growth of GO nanosheets, resulting in the decrease of bandgap energy. Chhowalla and co‐workers reported that the calculated gap between the highest occupied molecular orbital (HOMO) and the lowest unoccupied molecular orbital (LUMO) of a single benzene ring was 7 eV, while it decreased down to 2 eV for a cluster of 20 aromatic rings (**Figure** **2**A).^46^ Thus, the pure graphene nanosheet is a type of zero‐bandgap nanomaterial, but the gap energy will increase after cutting into ideal graphene quantum dots or clusters. At the typical sp^2^ domain size of 2.5–6 nm, the predicted bandgap in GO can be varied from 0.58 to 0.24 eV.^47^ Second, the oxygen‐containing groups, such as hydroxyl, epoxy, and carboxyl groups, interrupt the conjugated π system of GO nanosheets and isolate the sp^2^ carbon into independent graphene‐lattice islands. Therefore, the oxygen coverage in GO nanosheets would influence their bandgap energies. Huang et al. investigated the stability of GO bandgap for oxygen density from 6.25% to 50%,^48^ and found that the most stable bandgap for the lowest calculated coverage (6.25%) was 0.109 eV and for the highest calculated coverage (50%) was 3.004 eV (Figure 2B). Notably, the bandgap was not a monotonic function of oxygen density but exhibited local minimal values at O/C = 11.1% and 25%. Third, for ideal graphene clusters, the gap energy decreases after conjugation with various functional groups such as atomic vacancy, hydroxyl, epoxy, and carboxyl groups. Chien et al. found that these functional groups created distortions in the aromatic rings, and these disorder‐induced localized states caused the absorption in the lower energy regions based on Gaussian and the time‐dependent density functional theory simulations (Figure 2C).^49^

In brief, the precise chemical structures of GO have always been controversial during these years, and even to this day, no unambiguous model exists. The greatest divergence between every model is the types and locations of oxygen‐containing groups on GO nanosheets. However, the nonstoichiometry chemical structures, inhomogeneous morphologies, and random distributions of functional groups on GO greatly inspire researchers' interests on exploring its novel optical properties. From the viewpoint of making GO luminesce, it is necessary to modulate its bandgap through altering its morphology sizes or oxygen coverages according to its chemical structures. **Table** **1** summaries the reported strategies for size modulation or chemical modification of GO nanosheets, which were always used to synthesize luminescent GO nanomaterials.

**Table 1:**
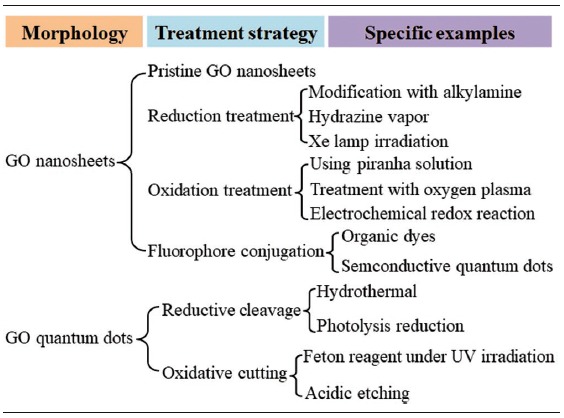
Summary of the reported strategies for the size modulation or chemical modification of GO nanosheets

## 3 Fluorescence from Diverse GO Nanosheets and Their Derivatives

In contrast to graphene with infinite sp^2^ system, GO contains a number of sp^3^ carbon atoms covalently bonded to oxygen, forming the isolated sp^2^ domains located at the large GO plane, as discussed above. Therefore, the HOMO and LUMO of GO are isolated with a definite bandgap in GO nanosheets, and the bandgap value increases with the oxygen coverage density and their different arrangements.^49^ In theory, a faint original fluorescence with very low quantum yields can be expected in common GO nanosheets synthesized by the classical methods, which is attributed to the π electrons confined in localized sp^2^ domains.^50^ With in‐depth understanding of luminescent behaviors of GO, a new field and bright future of luminescent GO nanomaterials have been shed by recent intensive explorations on synthesis, modification, and luminescent mechanism.

### 3.1 Fluorescence from Pristine GO Nanosheets

Enlightened by the fluorescence of single‐wall carbon nanotubes,^51^ the fluorescence of original GO was first reported by Dai and co‐workers in 2008. They developed a density gradient ultracentrifugation method to separate ultrasmall nano‐GO (NGO) with lateral dimensions down to 10 nm, and found that the NGO solution gave a PL emission peaked at 570 nm when excited at 400 nm.^52^ After modification with amine‐terminated branched polyethylene glycol (PEG), the obtained NGO–PEG exhibited an emission maximum blueshifted to 520 nm. After this initial study, many efforts have been made to investigate the PL features of small sized GO, which might be located at red regions. For example, Zhang et al. have prepared small and uniform sized GO (S‐GO) sheets by using carbon nanohorns as starting materials via an oxidative exfoliation method.^53^ The obtained S‐GO possessed fluorescence peak position at 560 nm when excited at 440 nm, but was gradually redshifted to 660 nm when excitation wavelength increased to 600 nm. For the large sized GO nanosheets, its original fluorescence also has been broadly reported. For instance, Luo et al. have reported a PL emission centered around 750 nm when excited at 500 nm for both aqueous and solid GO nanosheets.^54^ Chen and Yan also reported that GO colloid gave a NIR fluorescence with a maximum at 650 nm upon excitation at 450 nm, and believed it was presumably assigned to the radiative recombination of e–h pairs localized within small sp^2^ carbon domains embedded in sp^3^ matrix.^55^ This unique NIR fluorescence exhibited a reversible and sensitive response to ionic strength and pH values, and also was used to develop a biosensor for dopamine through photoinduced charge transfer mechanism.[qv: 18c] Likewise, Galande et al. have found strongly pH‐dependent visible fluorescence from aqueous dispersion of GO.^56^ The GO suspension showed a broad peak centered near 668 nm under 440 nm excitation. As pH increased from acidic toward neutral values, a monotonic decrease of this emission peak was observed. The emission at 668 nm disappeared and two relatively sharp peaks near 482 and 506 nm appeared after increasing pH values.

As to the origin of GO fluorescence, its electronic band structure and the density of states near Fermi level should be considered. Around this theory, a number of related hypotheses for luminescent mechanisms of pristine GO have been proposed. The theoretically calculation suggested a bandgap ranging from 0 to few electron volts, which was dependent on the ratio of carbon bonds to oxidation functional groups and their different arrangements.^57^ According to the band structure and density of state, Eda et al. ascribed the fluorescence of the as‐prepared GO to the optical transitions from structural disorder‐induced localized states in the π–π* gap of sp^2^ sites.^46^, ^48^ Gurzadyan and co‐workers believed that all three kinds of functionalized groups C—O, C=O, and O=C—OH were related to the emission.^57^ They found that the dominant fluorescence was found to originate from electronic transitions among nonoxidized carbon regions and boundary of oxidized carbon atom regions.

To explain the strong excitation wavelength dependent fluorescence of GO, Cushing et al. proposed a “giant red‐edge effect” mechanism.[qv: 58a] In their work, GO was chemically treated in HNO_3_ and KOH solutions to create carboxyl‐rich and hydroxyl‐rich GO, respectively. The carboxyl‐rich GO showed a broad emission band centered at 630 nm, but the hydroxyl‐rich GO exhibited at 500 nm. The fluorescence of primary GO was observed to be a superposition of these two emissions. When GO nanosheets were present in a polar solvent, the solvation dynamics were slowed down to the same time scale as the fluorescence, which broadened the fluorescence peak of GO and redshifted up to 200 nm with increasing of excitation wavelengths. This “giant red‐edge effect” of GO disappeared in a nonpolar solvent, leading to a narrow fluorescence peak that was independent of the excitation wavelength. In order to further discover what GO characteristics were responsible for the presence and extent of the giant red‐edge effect, they recently investigated the excitation wavelength–dependent fluorescence in a series of nitrogen‐, boron‐, fluorine‐, and oxygen‐doped GO, and found that only the out‐of‐plane strain was the critical factor in GO.[qv: 58b]

Moreover, a few researchers believed that the fluorescent properties of GO should be ascribed to some highly oxidized molecular species, OD, which adsorbed on GO nanosheets. Rourke and co‐workers have found that treatment of GO with NaOH separated the material into two components: a colorless but highly fluorescent OD and a darker nonfluorescent material containing GO‐like sheets.^59^ The as‐produced GO showed a weak and broad PL, while the OD fluoresced more intensely. Therefore, they believed that those OD adsorbents were accountable for the optical properties of GO. However, Naumov et al. recently reported that the unique spectroscopic properties of the combined system were independent on the presence of OD through analysis of absorption and emission spectra as well as lifetime measurements, while confirmed that the optical properties were governed by its internal structure rather than OD.^60^

### 3.2 Fluorescence from Functionalized GO Nanosheets

The oxygen‐containing groups on GO nanosheets, such as carboxyl groups or epoxy groups, always induce nonradiative recombination of e–h pairs, making the emission intensities of pristine GO nanosheets very weak. Therefore, many efforts have been devoted to improve GO luminescent efficiency through chemical functionalization of GO nanosheets to remove these sites. Our group found that butylamine‐modified GO nanosheets, through amide reaction of carboxyl groups and ring‐opening reaction of epoxy groups, showed an emission maximum at 430 nm under the excitation wavelength of 350 nm with a quantum yield of 13%, which was greatly enhanced relative to the original GO nanosheets.^7^ Similar PL characteristics could also be observed when GO was modified with other alkylamines such as 1,6‐hexylenediamine, octylamine, dodecylamine, and diamine‐terminated poly(ethylene glycol). Many other strategies have been developed to remove these nonradiative recombination sites, achieving bright luminescence, which are detailedly discussed in the following section.

#### 3.2.1 Reductive/Oxidative Treatment of GO Nanosheets

**Figure 3:**
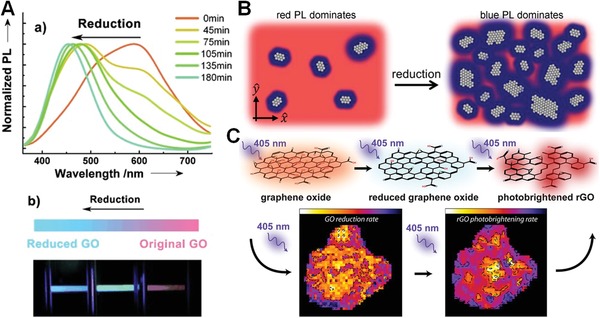
A) Tunable PL from GO. a) Normalized PL spectra of GO suspensions after photothermal reduction treatment. b) Photographs of GO suspensions at different reduction times. Reproduced with permission.^49^ Copyright 2012, Wiley‐VCH. B) Schematic illustration of ultrafast PL migration of GO after reduction. Reproduced with permission.^61^ Copyright 2013, American Chemical Society. C) Schematic illustration of direct observation of spatially heterogeneous single‐layer GO reduction kinetics. Reproduced with permission.^62^ Copyright 2013, American Chemical Society.

Reductive treatment is always used to remove oxygen‐containing groups during fabrication of graphene from GO nanosheets. Meanwhile, the bandgap structure of GO nanosheets is modulated, leading to many novel optical properties. For instance, Eda et al. observed blue PL centered around 390 nm for thin‐film samples deposited from thoroughly exfoliated suspensions after exposure to hydrazine vapor.^46^ By appropriately controlling the concentration of isolated sp^2^ clusters within the carbon–oxygen sp^3^ matrix through reduction treatment, the localizations of e–h pairs were facilitated to radiative recombination, and the PL intensity can be increased by tenfold compared to the as‐synthesized material. Moreover, it was found that bandgap energy of GO nanosheets can be continuously tuned by precisely controlling reductive extent, thus inducing diverse luminescent colors. Chen and co‐workers studied the PL evolution from GO to reduced GO by steady‐state Xe lamp irradiation with different exposure times.^49^ Incremental reduction times led to a gradual decrease in the red emission and a corresponding increase in the blue emission. The emission peak shifted from 600 to 450 nm (**Figure** **3**A). Kikkawa and co‐workers also found that the PL shifted from predominately red emission to blue after continuous wave xenon lamp exposure (Figure 3B).^61^ By the aid of characterization of fluorescence quenching and blueshift of the single GO sheet, McDonald et al. have directly observed the light‐induced photolysis of GO's oxygen containing functional groups (Figure 3C).^62^ Infrared irradiative reduction also has been used to investigate relationship between the modification of individual oxygen functional groups and the resultant optical properties of GO.^63^ All of these studies demonstrated that reductive treatment led to removal of oxygen‐containing groups, and creation of numerous small sp^2^ clusters within the sp^3^ matrix. The defect‐assisted localized states in GO due to oxygen‐containing groups induced longer wavelength emission, while the e–h recombination among these newly formed sp^2^ clusters gave out short wavelength emission. Therefore, the tunable fluorescence during reduction is attributed to the variation of the relative fluorescence intensity ratios from the heterogeneous electronic structures of GO.^48^, ^61^, ^62^, ^63^

Another interesting strategy is doping new luminescent center in GO nanosheets during solvothermal reduction process to achieve multicolored emission. Gan et al. reported that Mn^2+^‐bonded GO can exhibit tunable PL in the range of 400–550 nm.^64^ Authors dispersed GO and KMnO_4_ in deionized water, and then transferred the supernatant to an autoclave and heated to form Mn^2+^‐bonded reduced GO. The PL peak position monotonically increased from 430 to 550 nm as the excitation wavelength increased from 320 to 490 nm. The intensity of the long‐wavelength PL (480–550 nm) stemmed from the introduction of Mn^2+^ was enhanced more than the blue emission at 430 nm, which was essentially a quantum confinement effect related to the size of the sp^2^ clusters. Since Mn^2+^ was bonded to the sp^2^ clusters, the short distance and energy level overlap led to effective energy transfer from Mn^2+^ to the sp^2^ clusters. As a result, the radiative recombination rate was increased significantly and the PL was consequently enhanced.

**Figure 4:**
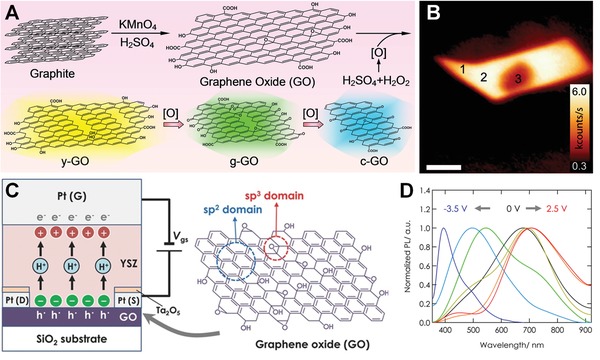
A) Schematic illustration for the preparation of multicolored GO nanosheets by reacting with atomic oxygen. Reproduced with permission.^21^ Copyright 2016, American Chemical Society. B) Confocal PL image for a graphene nanosheet after treating with oxygen plasma for 5 s. Reproduced with permission.^65^ Copyright 2009, American Chemical Society. C) Schematic illustration of GO‐based all‐solid‐state electric double‐layer transistor. G, S, and D denote the gate, source, and drain electrodes. Reproduced with permission.[qv: 66a] Copyright 2014, Wiley‐VCH. D) Direct current bias dependence of normalized PL spectra tuned by application of various bias voltages for 1200 s. Reproduced with permission.[qv: 66b] Copyright 2015, American Chemical Society.

Many oxidative protocols also have been reported for tuning the bandgap structure of GO nanosheets to achieve diverse fluorescence colors. Our group recently reported a novel method to synthesis multicolored emissive GO nanosheets.^21^ We found that the emissions of GO can be tuned from dark brown to cyan with emission peaks shifting from 590 to 490 nm, simply through modulating oxidation times in piranha solution (**Figure** **4**A). This phenomenon was ascribed to atomic oxygen generated in piranha solution, which could convert carbon double bond in aromatic rings of GO nanosheets into epoxy groups, then hydroxyl groups or carbonyl pairs. Therefore, with elongating reaction time, the size of sp^2^ carbon cluster decreased, leading to the extension of π→π* energy gap and the emissions blueshift. GO obtained from single layer graphene sheet by short time treatment with oxygen plasma could also exhibit bright emission with a broad band centered around 700 nm (Figure 4B).^65^ Intense laser excitation with power exceeding 1 mW would lead to the attenuation of emission intensities (position 3 in Figure 4B). For multilayer graphene sheets, no luminescence could be observed following this treatment, which because only the topmost layer was etched by oxygen plasma and the formed emission could be immediately quenched by subjacent untreated graphene layers.

With the help of redox reaction using a solid electrolyte thin film, the bandgap of GO can be in situ and nonvolatile tuned by simply applying direct current (DC) voltage within an all‐solid‐state device (Figure 4C).^66^ The PL peak wavelength can be tuned from 393 to 712 nm by adjusting the applied DC voltage between −3.5 and 2.5 V (Figure 4D), where the polarity of voltage was defined as positive when GO was oxidized and negative when reduced. For the pristine GO, a broad emission peak at 676 nm was observed. This red emission became weak and another peak appeared at 544 nm upon a DC bias of −2.5 V. Upon subsequent application of an opposite DC bias voltage (1.5 V), an electrochemical oxidation from reduced GO to GO was caused, and the PL peaks can shift from 544 to 690 nm.

#### 3.2.2 Fluorescence from Fluorophore‐Conjugated GO Nanosheets

Direct conjugation of fluorophores onto GO nanosheets always failed to endow it fluorescent ability because of resonance energy transfer mechanism. However, a few ingenious designs could anchor the fluorescein on GO nanosheets and effectively prevent fluorescence quenching. For example, Huang and co‐workers have reported that PEG2000 was covalently grafted to hydroxyl and carboxylic groups of GO.^67^ Fluorescein (Fluo) was then activated and further conjugated to PEG‐modified GO (Fluo‐G). PEG served as a bridge between fluorescein and GO sheets, and prevented fluorescence quenching. Compared with the spectrum of free fluorescein, a redshift of 5 nm was observed in the spectrum of Fluo‐G, suggesting the successful conjugation. The approach of direct binding of semiconductors QDs to GO also failed to make it fluoresce.^68^ To overcome this problem, Hu et al. modified GO by the surface adsorption of an amphiphilic polypeptide, poly(l‐lysine), which had functions of improving dispersion and endowing good adhesiveness to GO surface.[qv: 69a] After overnight incubation, QDs were adsorbed to the polypeptide–GO. The obtained QD–GO nanocomposites were highly stable and showed a bright fluorescent image from a glass slide that contained a spread mixture of three differently colored QD–GO samples under irradiation from a single UV source. Additionally, the PL of QD–GO nanocomposites could be used for in vivo imaging of internal tissues in small animals. Zhu et al. used NGO as a surface modification agent to encapsulate a type of aggregation‐induced emission nanoparticle (NP) (TPE‐TPA‐FN, TTF).[qv: 69b] The surface modification of NGO not only increased the emission efficiency of TTF nanoparticles, but also improved their water dispersibility. With the help of high nonlinear optical efficiency of TTF, the obtained TTF–NGO NPs could emit bright three‐photon luminescence, and were further used to image the architecture of blood vessels in mice ears and the distribution of nanoparticles in zebrafish.

### 3.3 Fluorescence from GO Quantum Dots

In order to effectively modulate bandgap structure to achieve diverse fluorescence of GO, another promising approach is to cut and exfoliate the nanosheets into smaller and thinner 0D QDs, because this variation can greatly increase the number and density of isolated sp^2^ domains. On the other hand, the small size and high water dispersibility of GO QDs can also greatly promote its cellular entrance for biological applications. The fluorescent GO QDs reviewed herein mainly focused on the materials obtained from GO nanosheets by means of reduction cutting or oxidation cutting. Novel luminescent phenomena different from traditional GO nanosheets are associated with the quantum confinement and edge effects occurring in GO QDs.

#### 3.3.1 Reductive Cleavage of GO Nanosheets into QDs

**Figure 5:**
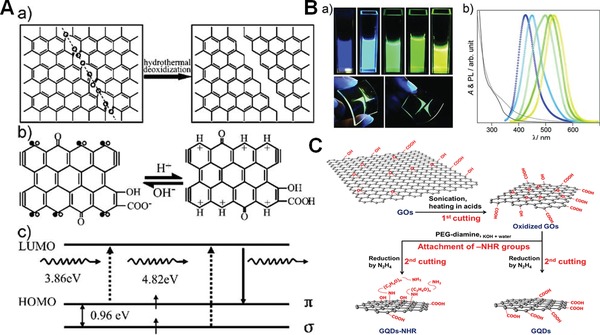
A) Hydrothermal reduction of GO nanosheets into blue luminescent graphene QDs. a) Mechanism for the hydrothermal cutting of GO into GQDs. b) Models of the GQDs in acidic (right) and alkali (left) media. c) Typical electronic transitions of triple carbenes at zigzag sites. Reproduced with permission.^70^ Copyright 2010, Wiley‐VCH. B) Optically tunable amino‐functionalized graphene QDs. a) Emission images of the obtained GQDs (upper) and GQD@polymer hybrids (bottom) under irradiation with a 365 nm UV lamp. b) PL and selected UV–vis absorption spectra of the obtained GQDs. Reproduced with permission.^73^ Copyright 2012, Wiley‐VCH. C) Scheme for preparation of GQDs and GQDs–NHR from GO. Reproduced with permission.[qv: 74a] Copyright 2013, American Chemical Society.

To the best of our knowledge, top‐down method is widely used to prepare GO‐based QDs, such as solvothermal means, electrochemical cutting methods, and so on. Pan et al. reported a simple hydrothermal approach for the cutting of micrometer‐sized GO sheets into surface functionalized QDs with the average diameter of 9.6 nm for the first time.^70^ The obtained QDs emitted bright blue luminescence at 430 nm with a quantum yield of 6.9% when excited at 320 nm. They proposed that the epoxy groups and carbonyl groups in GO nanosheets broke up during the hydrothermal deoxidization process to form QDs eventually, and generate free zigzag sites with a carbene‐like triplet ground state accounting for the strong blue fluorescence (**Figure** **5**A). Kim et al. also used hydrothermal cutting method to prepared a series of particular sized GO QDs for studying relationship between edge shapes and fluorescence properties.^71^ They reported that these QDs appeared as the circular shape with mixed edges of zigzag and armchair when its diameter was lower than 17 nm, but as the polygonal shape mostly with armchair edges for diameters larger than 17 nm. With varying the average size of QDs from 5 to 35 nm, the visible PL peak energy showed a minimum when the diameter was 17 nm. This unusual PL behaviors were ascribed to the circular‐to‐polygonal‐shape and the corresponding edge‐state variations as the QD sizes increased. Besides solvothermal cutting method, photolysis reduction could also cut GO nanosheets into QDs. Sun et al. used isopropanol as the reducing agent assisted with UV irradiation to prepare bright blue luminescent GO‐based QDs.^72^ When irradiated by UV light, (CH_3_)_2_C• was yielded from isopropanol, which could reduce the oxidative groups on GO nanosheets to realize photochemical reduction and cutting. These studies demonstrated that the disorder states induced by oxygen‐containing groups decreased and large number of isolated sp^2^ domains formed after reductive cutting, and the e–h recombination among these sp^2^ cluster‐like states greatly enhanced the fluorescence.

Despite many efforts have been made for the synthesis of GO‐based QDs, most of them exhibit similar blue fluorescence with the maximum emission peak centered around 420 nm. It is intriguing if the bandgap energy can be continuously modulated to achieve multicolored emission after cutting into QDs. Tetsuka et al. recently reported a novel hydrothermal pathway for graphene‐based QDs (GQDs) from GO nanosheets that was edge terminated by a primary amine, allowing the electronic structure to be modified through the effective orbital resonance of amine moieties with graphene core.^73^ The fluorescence of GQDs could be tuned from violet to yellow region simply by changing the initial concentration of ammonia and the temperature of the amino hydrothermal treatment (Figure 5B). A primary amine at the edge significantly altered the whole electronic structure of the GQDs, resulting in narrowing the optical bandgap. The resonance feature between the delocalized π orbital and the molecular orbital in the amino group was thus an origin of both the edge treatment dominated optical tunability and the high quantum efficiency. Many other groups also found that different amino‐groups' modification of QDs edge structure would induce different colored fluorescence. For instance, Jeon and co‐workers have also compared the fluorescence of GO‐based QDs with and without amine functional groups (Figure 5C).^74^ In their work, GO was first cut by oxidation in a mixture of H_2_SO_4_/HNO_3_, and then further reduced by a N_2_H_4_ reduction process. To fabricate the amine group–functionalized GQDs (GQD–NHR), oxidized GO was reacted with diamine terminated polyethylene glycol (PEG‐diamine) before N_2_H_4_ reduction steps through the ring‐opening reaction of the epoxy groups. The obtained GQDs exhibited a bluish‐green color with the maximum PL position at 500 nm, whereas the GQD–NHR exhibited a more yellowish color with emission peak red‐shifted to ≈528 nm. Wu et al. recently also demonstrated that GQDs exhibited PL emission from blue to yellow after a one‐pot hydrothermal treatment of nanosized GO with ammonia, aliphatic primary amines, and amino‐substituted organelle targetable compounds, respectively.^75^

#### 3.3.2 Oxidative Cutting of GO Nanosheets to QDs

**Figure 6:**
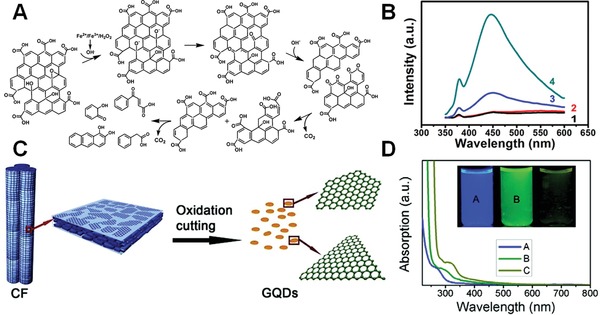
A) Scheme for the photo‐Fenton reaction of GO sheets. B) The corresponding PL spectra of the GO aqueous suspensions before and after reactions for different times. Reproduced with permission.^76^ Copyright 2012, American Chemical Society. C) Scheme for oxidation cutting of carbon fiber into GQDs. D) UV–vis spectra and corresponding fluorescent photos of GQDs after reacting at different temperatures. Reproduced with permission.^77^ Copyright 2012, American Chemical Society.

Oxidative cutting is another effective method for preparation of GO‐based QDs. The formed QDs are also expected to exhibit excellent fluorescence properties due to the quantum confinement and edge effects. Zhang and co‐workers demonstrated an oxidative strategy to cut micrometer‐sized GO sheets by Fenton reagent under an UV irradiation (**Figure** **6**A).^76^ The photo‐Fenton reaction of GO was initiated at the carbon atoms connected with oxygen containing groups, and C—C bonds were broken subsequently. The as‐generated QDs have uniform crystallinity because the carbon atoms connected with the hydroxyl and epoxide groups have been removed during the photo‐Fenton reaction, and assumed the strong fluorescence centered at 450 nm with an excitation‐dependent emissive property (Figure 6B). Peng et al. also have reported an acidic etching method to cut traditional pitch‐based carbon fibers into QDs in the size range of 1–4 nm (Figure 6C).^77^ The fluorescence of QDs can be tailored to blue, green, and yellow by varying the size of QDs, which should be ascribed to the variation in density and nature of sp^2^ sites (Figure 6D). Tour and co‐workers have developed a facile approach to prepare different nanometer‐sized QDs from various coals.^78^ The GQDs derived from bituminous coal named as b‐GQDs exhibited diameters of 2.96 ± 0.96 nm. GQDs from coke (c‐GQDs) and anthracite (a‐GQDs) showed a uniform size of 5.8 ± 1.7 and 29 ± 11 nm. The emission maxima of a‐GQD, c‐GQD, and b‐GQD solutions were at 530, 480, and 460 nm when excited at 345 nm, respectively. Additionally, the bandgap of the GQDs synthesized from anthracite could also be controlled by the reaction temperature of oxidation process, resulting in the synthesis of different sized GQDs with fluorescence properties covering the visible spectrum. As expected, when the GQD size increased from 4.5 to 70 nm, the peak emission was redshifted from 520 to 620 nm, which was in accordance with the quantum confinement effect. Li et al. have reported an alternative electrochemical approach for direct preparation of GO‐based QDs with a uniform size of 3–5 nm, which presented a green luminescence and could be retained stable in water for several months.^79^

#### 3.3.3 Luminescence Difference between GO‐Based QDs and Graphene QDs

**Figure 7:**
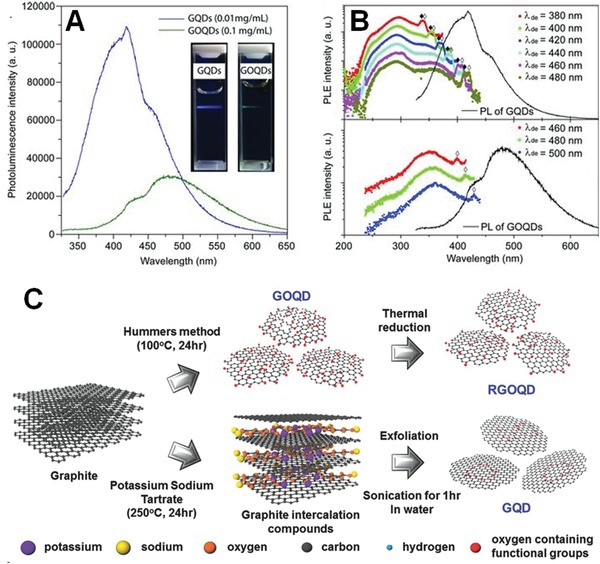
A) PL spectra and the corresponding fluorescent images of GQDs and GOQDs. B) PL excitation spectra of GQDs and GOQDs with varying detection emission wavelengths. Reproduced with permission.^80^ Copyright 2013, Wiley‐VCH. C) Schematic illustration of entire processes for GOQD, reduced GOQD (RGOQD), and GQD. Reproduced with permission.^81^ Copyright 2016, Wiley‐VCH.

The detailed investigation of fluorescence difference between QDs directly derived from graphene (GQDs) and QDs obtained from GO (GOQDs) can benefit the in‐depth understanding of their luminescence mechanism. For example, Liu et al. have successfully synthesized highly homogeneous GQDs by shaking graphite nanoparticles in an ethanol/H_2_O mixture on a vortex mixer, and GOQDs from the suspension of GO solution after sonication and centrifugation.^80^ Both of the obtained GQDs and GOQDs exhibited circular shapes within a 4 nm diameter, however, GOQDs possessed various oxygenous functional groups, and GQDs had pure sp^2^ carbon crystalline structure without oxygenous defects. The spectral study showed that GQDs and GOQDs exhibited clear blue (420 nm) and green color (480 nm) emissions, respectively, and the fluorescence intensity of GQDs was about 3.5 times higher than that of GOQDs, although its concentration was about 10 times smaller than that of GOQDs (**Figure** **7**A,B). Yoon et al. also employed graphite as a starting material to synthesize GOQDs, reduced GOQDs, and GQDs (Figure 7C), and then systematically investigated their fluorescence difference.^81^ It was found that the fluorescence intensity was remarkably quenched and the emission peaks were redshifted and broadened after increasing the oxygen concentration. Therefore, these luminescence differences revealed that the blue luminescence of GQDs was dominated by intrinsic states in the high‐crystalline structure, but the green luminescence of GOQDs originated from defect states with oxygenous functional groups.^80^, ^81^

## 4 Unusual Luminescence Behaviors from GO

Although various photoluminescence behaviors have been revealed on GO nanosheets and QDs, these advances are mainly based on our current knowledge of the structures and compositions of GO, as well as the inspirations from other luminescent material systems. Actually, GO nanosheets also exhibit many intriguing luminescent properties, such as multiphoton luminescence, electroluminescence, and chemiluminescence. In this part of review, we summarized the latest discoveries about these unusual luminescence ascribed to the synergetic interactions of quantum confinement and surface chemistry effect.

### 4.1 Multiphoton Fluorescence from GO

Since Li and co‐workers reported the upconversion luminescence (UCL) phenomenon of hydrazine hydrate reduced GO–PEG,^82^ several publications have reported that the UCL occurred in various GO derivatives. For instance, alkylamine‐modified GQDs exhibited an upconversion emission wavelength redshift when changing excitation from 600 to 900 nm, which was deduced to the two or multiphoton active process.[qv: 83a] Li et al. reported a hydrothermal approach for preparation of N‐doped GQDs with a diameter of ≈1–7 nm, which also possessed excellent UCL properties.[qv: 83b] The upconverted emission peaks shifted from 415 to 516 nm when excitation wavelengths changed from 560 to 900 nm. The maximum PL intensity appeared at 425 nm when excitation wavelength was 640 nm. However, as many literatures reported, an intermediate state is essential for the upconversion of the carriers and the excitation light should be coherent photons from a laser. Tan et al. recently commented that there might be mistakes in interpreting normal luminescence excited by the second‐order diffraction light of wavelength λ/2 as evidence of UCL.^84^ Gan et al. also observed that the GQD's emission intensity dependent on temperature and excitation intensity exhibited the same features as those from normal PL.^85^ The time resolved PL confirmed that the decay of the emission obtained with 640 nm excitation was almost equal to the emission obtained with 320 nm excitation. All the results indicated that the so called “UCL” under excitation of a xenon lamp in GQDs was artificial, and should be contributed to the second‐order diffraction light of wavelength λ/2 coexisting in the excitation light of wavelength λ. In addition, they also observed the real UCL from GQDs under excitation of a pulsed laser.

Li et al. developed GO nanoparticles with the maximum emission peak located at 590 nm for two‐photon luminescence cell imaging.^86^ GO nanoparticles were functionalized with transferrin (Trf) and PEG molecules for targeting cancer cells and stabilizing the particles in cell culture buffers, respectively. A plot of the PL intensity versus the incident power on a logarithmic scale fitted a straight line with a slope close to two, indicating that the PL was attributed to two‐photon excitation. A low power with 7 mW of an ultrafast pulsed laser was sufficient to induce strong PL in living cells, while 30 mW was needed for Trf‐modified fluorescein isothiocyanate (FITC) dyes. Almost at the same time, He and co‐workers also observed the two‐photon‐ and three‐photon‐induced PL from GO nanoparticles under femtosecond (fs) laser excitation.[qv: 87a] A two‐photon luminescence spectrum was observed in the range of 400–700 nm with the maximum peak located at 550 nm by using a 810 nm fs pulsed laser. Authors found that the two‐photon‐induced emission intensity was proportional to the square of the fs excitation intensity, confirming the two‐photon process. Similarly, the three‐photon luminescence was also observed when GO sample was stimulated by 1260 nm pulse laser. After conjugation with PEG molecules, the GO nanoparticles were intravenously injected into mice body from the tail vein, and observed their flow, distributions, and clearance in the blood vessels by utilizing a deep‐penetrating two‐photon imaging technique. Moreover, GO–PEG nanoparticles were also microinjected into the brain of gene‐transfected mice, and showed that the nanoparticles located at 300 µm depth in the brain could be clearly distinguished. Recently, they also reported an upconversion luminescence of GO nanosheets in a GO hybrid waveguide system under excitation by the continuous‐wave laser.[qv: 87b] Gong and co‐workers also reported that the N‐doped GQDs (N‐GQDs) prepared by a facile solvothermal method using dimethylformamide as a solvent and nitrogen source, possessed two‐photon luminescent properties.^88^ A low laser power of 1 mW was sufficient to excite strong two‐photon fluorescence of N‐GQD in this work. More importantly, a large imaging depth of 1800 µm was achieved in tissue phantom using N‐GQD as two‐photon probes, which significantly extended the fundamental two‐photon imaging depth limit.

### 4.2 Electroluminescence of GO Derivatives

The high carrier mobility and thermal conductivity of graphene facilitate the spatially localized accumulation of hot electrons in an electrically biased suspended graphene layer, making graphene an ideal material to serve as a nanoscale thermal radiative emitter. For instance, Freitag et al. demonstrated a thermal mid‐infrared emission from electrically biased graphene on SiO_2_ substrates.[qv: 89a] However, such thermal radiation was inefficient, and only a small fraction of the applied energy was converted into light radiation. To overcome this problem, Kim et al. developed an electrically biased suspended graphene device, and found that the suspended graphene channel began to emit visible light at its center once the source–drain bias voltage exceeded a threshold value, and the emission brightness and area increased with bias voltage.[qv: 89b] The thermal radiation efficiency was enhanced to 1000‐fold, and the emitted light was intense to be visible by the naked eye.

**Figure 8:**
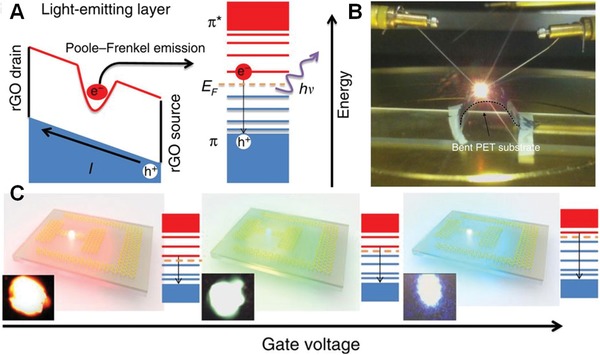
A) Scheme for the charge injection process of GFLED. B) Bright red emission from the GFLED on a flexible polyethylene terephthalate substrate. C) Schematic of the gate voltage‐dependent EL. Inset photos were corresponding emission images from a real device. Reproduced with permission.[qv: 90b] Copyright 2015, Springer Nature.

Except for thermal radiation, graphene also could act as an EL material in nanoscale field‐effect light‐emitting diode (LED) with tunable emission color.[qv: 90a] For example, Ren and co‐workers have demonstrated a bright spectrally tunable EL from blue (450 nm) to red (750 nm) at the reduced GO‐based field‐effect LED (GFLED), as shown in **Figure** **8**.[qv: 90b] They explained that the EL resulted from the recombination of Poole‐Frenkel emission ionized electrons at the localized energy levels arising from semireduced GO, and holes from the top of π band (Figure 8A). The GFLED device showed a high brightness of up to 6000 cd m^−2^, with efficiency around 1%, and the EL emissions were continuously tuned from light blue to dark red by electrically or chemically adjusting the Fermi levels (Figure 8C). Under high field, the localized electrons were excited into the lowest unoccupied discrete energy level. Radiative recombination between the thermally emitted electrons and the free holes resulted in the EL. Additionally, graphene or graphene quantum dots ranging from 2 to 10 nm also could be used to prepare LED devices for achieving bright EL.^91^

Intense electrogenerated chemiluminescence (ECL) signals of GO also have been reported by many groups. For example, Fan et al. have reported fairly intense ECL which was over than 1.8 × 10^6^ photon counts s^−1^ cm^−2^ from a GO aqueous suspension containing NaClO_4_, phosphate buffer saline, and tri‐*n*‐propylamine (TPrA).^92^ When the electrode potential was scanned to a positive value, further oxidation of GO directly took place either on the Pt electrode during collision or via TPrA radical cations. The deprotonation reaction of TPrA generated a highly reductive radical intermediate and the radiative recombination of this radical and GO led to the formation of excited‐state of GO for the generation of ECL. Likewise, Zhu and co‐workers also found the ECL phenomenon from GO‐derived GQDs.^93^ They developed a facile one‐pot microwave‐assisted approach for the preparation of GQDs with greenish‐yellow PL from GO nanosheets under acid conditions. Furthermore, the GQDs were moderately reduced with NaBH_4_ to obtained blue‐luminescent GQDs. Except for well‐known PL properties, these two kinds of GQDs behaved intensive ECL with K_2_S_2_O_8_ as coreactant. They believed that the ECL mechanism of the GQDs was similar to that of semiconductor QDs, Si nanoclusters, and carbon nanoclusters. First, strongly oxidizing SO_4_^•−^ radicals and GQDs^•−^ radicals were produced by electrochemical reduction of S_2_O_8_^2−^ and GQDs, respectively. Then, SO_4_^•−^ radicals could react with GQDs^•−^ via electron‐transfer annihilation, producing an excited state that finally emitted light.

### 4.3 Chemiluminescence Initiated by GO

**Figure 9:**
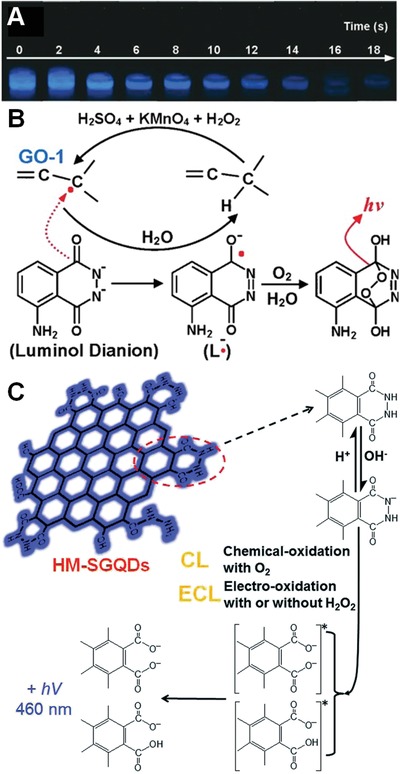
A) The strong and long‐lasting visible CL produced by addition of luminol into freshly prepared GO solution. B) The CL mechanism triggered by the π‐conjugated carbon radicals in GO. Reproduced with permission.^22^ Copyright 2014, Wiley‐VCH. C) CL mechanism of hydrazide‐modified single‐layer GQDs. Reproduced with permission.[qv: 97a] Copyright 2014, Royal Society of Chemistry.

Similar as fluorescence quenching, GO also could quench the CL through resonance energy transfer (CRET) mechanism.^94^ For instance, Park and co‐workers recently designed a GO‐based CRET platform for homogeneous immunoassay of C‐reactive protein, which was a key marker for human inflammation and cardiovascular diseases in human serum samples.^95^ In the CRET platform, GO played a key role as an energy acceptor, while luminol served as a donor.^96^ Nevertheless, our group reported a novel phenomenon that the freshly prepared GO could directly trigger the CL of luminol without the need of additional oxidizing agents (**Figure** **9**A). We believed that excess H_2_O_2_ might be decomposed to ·OH radicals in the presence of KMnO_4_, and ·OH radicals immediately added to the double bonds at the GO plane, leading to form π‐conjugated carbon radicals (Figure 1C). As the carbon radicals were located at the π‐network plane, the single electron was likely to conjugate with π electrons at neighboring double bonds. The single electron was mobile among the conjugated C=C bonds, significantly prompting its oxidizing capability and the reaction efficiency with the target molecules adsorbed on the basal plane of GO, such as luminol molecules (Figure 9B). In contrast to previously reported results, the π‐conjugated carbon radicals directly initiated the long‐lasting, visible CL of luminol, which was even stronger than that triggered by horseradish peroxidase.^22^ This work provided a new insight into the structure and properties of freshly prepared GO.

Compared with large sized GO, decreasing its lateral dimension to less than 20 nm would greatly enhance the CL signals. For example, Chi and co‐workers recently reported that hydrazide‐modified single layer graphene quantum dots (HM‐SGQDs) prepared from GO, which had an abundant of luminol‐like units, accordingly exhibited unique and excellent CL in the absence of any strong oxidant. As shown in Figure 9C, the hydrazide group of HM‐SGQDs was deprotonated in basic solution to form an anion.[qv: 97a] Subsequently, the anions were chemically oxidized by the dissolved oxygen (O_2_), producing a strong CL signal. The CL intensity was mainly dependent on pH value and concentration of O_2_, implying the potential applications of HM‐SGQDs in pH and O_2_ sensors. Gao et al. also reported that immobilization of *N*‐(aminobutyl)‐*N*‐(ethylisoluminol) (ABEI) molecules onto the surface of GQDs demonstrated an excellent CL activity.[qv: 97b] This novel GQD platform showed nearly 7 times higher CL emission compared to that of ABEI‐functionalized GO nanosheets under the same conditions. Authors believed that the excellent CL property should be ascribed to the catalytic effect of GQDs, which facilitated the generation of several radicals to produce the excited state oxidation product of ABEI, resulting in strong light emission.

## 5 Conclusion and Outlook

Recent booming advances on synthesis of GO nanomaterials greatly promote the understanding of its chemical structures. It has been widely recognized that GO possesses various oxygen‐containing functional groups, such as hydroxyl and epoxy groups on the lateral plane, phenol and carboxylic groups at the edge of nanosheets. Some specific groups, such as ketone, quinone, five‐/six‐membered ring lactols, and even π‐conjugated carbon radicals vary for every study which is highly dependent on the synthesis conditions. This structural heterogeneity does not hamper GO applications on materials or chemistry fields, but endows GO with a multitude of unique properties including excellent water dispersibility, superior biocompatibility, and superior surface modifiability, particularly, its tunable luminescence properties. In this review, we systematically summarized the latest works about the various luminescence from GO and its derivatives, including PL, EL, and CL. After various chemical treatments, such as solvothermal reactions, reductive or oxidative cutting, surface modification, and so on, the morphology sizes or surface functionalities of GO would be kaleidoscopic, resulting in diverse luminescent signals. Upon summary of these advances, it can be observed that control of oxygen coverage (or the ratio of sp^2^ carbon to sp^3^ carbon) and the types of oxygen‐containing groups on GO nanosheets is crucial for enhancing the luminescent efficiency. In addition, the modulation of size and edge states (shapes or functional groups) is useful for tuning luminescence colors of GO nanomaterials. This principle provides significant guidance for the future design and synthesis of luminescent GO nanomaterials.

The developments of luminescent GO have been far‐reaching, however, there still remains several key challenges to improve its luminescent properties and expand their application boundaries. First, the nonstoichiometric chemical structures of GO make it difficult to precisely and accurately modulate its bandgap for obtaining specific fluorescence emissions. As depicted in the above sections, these luminescent GO nanomaterials commonly are a mixture of nanosheets with different sizes, resulting in broad emission bands. The complexity of GO samples tremendously limits their applications when employing its PL, CL, or ECL as signal donors. Therefore, achievement of controllably synthesis of uniform GO nanomaterials with high quantum yields just as semiconductor quantum dots and silicon nanocrystals, or purification their compositions through column chromatography, would greatly improve their luminescent properties. Second, the synthesis protocols which most literatures reported always lead to generate blue fluorescent GO nanomaterials, while precisely tuning luminescent colors to green, yellow, or red is rarely reported. A few latest works on fluorescent carbon dots demonstrated that the codoping of nitrogen, sulfur, or other heteroatoms in the luminescent center would extend emissions to near‐infrared region, which may offer a novel avenue to redshift GO luminescence.^98^ Third, the in‐depth luminescence mechanism should be further studied by using computational simulation and some specific photophysical measurements, such as synchrotron radiation technique and luminescent lifetime measurements under extreme conditions, which may reveal some novel luminescent properties, and provide significant guidance on the synthesis of high‐quality luminescent GO nanomaterials. In addition, this achievement can also suggest some advices on the preparation of other luminescent 2D nanosheets, such as black phosphorus, MoS_2_, etc., and elucidation luminescent mechanisms for these nanomaterials. Finally, bright EL of GO has been widely reported, yet seldom applied in biomedical sensing fields. Along with the rapid developments of flexible electronics, EL‐based wearable GO biomedical devices will become a promising direction because of its intriguing optoelectronic properties.

GO nanosheets, as a bridge of inorganic nanomaterials and organic molecules, are attracting increasing attention due to their intriguing physicochemical properties. It is anticipated that further combination of newly explored GO with many other functionalized nanomaterials, such as other 2D nanosheets, metal–organic frameworks, polymer nanoparticles, upconversion nanoparticles, aggregation‐induced emission nanoparticles, and so on,^99^ will provide new insights for constructing integrated platforms for biomedical, environmental, or energy applications.

## Conflict of Interest

The authors declare no conflict of interest.
